# Determinants for choice of home birth over health facility birth among women of reproductive age in Tanzania: an analysis of data from the 2015-16 Tanzania demographic and health survey and malaria indicator survey

**DOI:** 10.1186/s12884-020-03266-3

**Published:** 2020-09-24

**Authors:** Fabiola V. Moshi, Christopher H. Mbotwa

**Affiliations:** 1 Department of Nursing and Midwifery, College of Health Sciences, the University of Dodoma, P.O. Box 259, Dodoma, Tanzania; 2 Mbeya College of Health and Allied Sciences, University of Dar es Salaam, P.O. Box 608, Mbeya, Tanzania

**Keywords:** Childbirth, Home, Health facility, Women, Reproductive Age, Tanzania

## Abstract

**Background:**

While evidence has shown an association between place of birth and birth outcomes, factors contributing to the choice of home birth have not been adequately investigated in Tanzania while more than 30% of deliveries occur outside of health care facilities, and more than 95% of those deliveries are assisted by non-medical providers who are often unskilled. The use of unskilled birth attendants has been cited as a factor contributing to the high maternal and neonatal mortalities in low-resources countries. This study aimed to identify determinants of choice for home birth over health care facility birth in Tanzania.

**Method:**

This study used the 2015-16 Tanzania Demographic and Health Survey and Malaria Indicator Survey (2015-16 TDHS-MIS) dataset. A total of 2286 women of reproductive age (15–49 years) who gave birth within one year preceding the survey were included in the analysis. Both univariate and multivariable regression analyses were used to determine predictors for the choice of home-based childbirth over health care facility delivery.

**Results:**

A total of 805 (35.2%) women had a home birth. After adjusting for confounders, the determinants for choice of home birth were: the level of education (primary education [AOR = 0.666; *p* = 0.001]; secondary and higher education [AOR = 0.417; *p* < 0.001]), in reference to no formal education; not owning a mobile phone (AOR = 1.312; *p* = 0.018); parity (parity 2–4 [AOR = 1.594; *p* = 0.004], parity 5 and above [AOR = 2.158; *p* < 0.001] in reference to parity 1); inadequate antenatal visits (AOR = 1.406; *p* = 0.001); wealth index (poorest (AOR = 9.395, *p* < 0.001); poorer (AOR = 7.701; *p* < 0.001); middle (AOR = 5.961; *p* < 0.001); richer (AOR = 2.557; *p* < 0.001)] in reference to richest women; and Zones (Southern Highlands, [AOR = 0.189; *p* < 0.001]; Southern, [AOR = 0.225; *p* < 0.001]; Zanzibar, [AOR = 2.55; *p* < 0.001]) in reference to Western zone.

**Conclusions:**

A large proportion of women birth at home. Unskilled providers such as traditional birth attendants (TBAs), relatives or friends attend most of them. Predictors for home-based childbirth included lack of formal education, poor access to telecommunication, poor uptake of antenatal visits, low socio-economic status, and geographical zone. Innovative strategies targeting these groups are needed to increase the use of health care facilities for childbirth, thereby reducing maternal and neonatal mortality in Tanzania.

## Background

Maternal mortality remains a public health problem worldwide. In 2015, maternal mortality was the second leading cause of mortality among women of reproductive age worldwide [1]. Ninety four percent of these deaths occurred in developing counties, of which African countries accounted for 65% [1]. Tanzania has a high burden of maternal mortality with an estimated maternal mortality ratio of 546 deaths per 100,000 live births [2]. Most of these deaths are preventable if women receive appropriate antenatal and intrapartum care by skilled attendants [3]. Direct causes of maternal mortality contribute to up to 80% of all maternal deaths. These include severe bleeding usually after childbirth (postpartum hemorrhage), high blood pressure during pregnancy and the postpartum period (preeclampsia and eclampsia), and infection usually after childbirth or complications of abortion [1].

A ten-year retrospective study undertaken in Tanzania reported that the leading cause of maternal mortality was eclampsia (34.0%), followed by obstetric haemorrhage (24.6%) and maternal sepsis (16.7%) [4]. The main indirect causes of maternal mortality were anaemia (14.9%) and cardiovascular disorders (14.0%). The study also reported an increasing trend of maternal death due to haemorrhage and cardiovascular disorders during the study period [4]. Two-and-a-half million neonates died in 2017 worldwide [3]. Sub-Saharan Africa and South Asia were big contributors to global neonatal mortality [3]. Tanzania is among the sub-Saharan countries contributing to this rate, with an estimated 25 deaths per 1000 live births [2].

The risk of a woman to die from maternal causes in developing countries is high compared to developed countries (one maternal death for every 41 live births compared to one maternal death for every 3,300 live births respectively) [1]. A collective effort is needed to reduce the gap between the global north and the global south. Learning from developed countries is a cornerstone strategy towards decreasing the risk of maternal and neonatal mortalities in the developing countries. One of the strategies used by the developed countries is the use of skilled attendants to assist women during labour and childbirth. Almost all births in these regions are assisted by skilled birth attendants regardless of the place of birth [1]. In Tanzania, more than 30% of all births occur out of health facilities, and 96% of those births are assisted by unskilled providers such as traditional birth attendants (TBAs) or relatives or friends [2]. These birth attendants often lack the necessary skills to identify signs of complications. In most cases, there is a delay on referrals to the facilities resulting to morbidity and mortality among mothers and newborns [5].

Skilled birth attendance is defined as the process by which a delivering woman is provided with satisfactory care during labour, delivery and the early postpartum period by a trained health care provider [6]. Although the use of skilled birth attendants have shown to be an effective strategy towards the reduction of maternal and neonatal mortality, still a number of women in Tanzania end up birthing at home, where they hardly get assistance from skilled birth attendants. These birth attendants in Tanzania play an important role in reduction of maternal and neonatal mortality through maternal and neonatal health counseling and escorting woman in labor to health facility for skilled birth [7, 8]. Based on local regulations, TBAs in Tanzania are not officially allowed to assist birth. However, they are allowed to provide maternal reproductive health counselling and escorting a woman to a health facility [7, 8]. Unlike developed countries, Tanzania lacks trained birth attendants who can assist delivery outside health facilities. Hence, most births occurring outside the health facilities are assisted by unskilled providers. In this study, home birth refers to all births which occur outside health facilities including but not limited to births at home and TBA clinics.

The World Health Organization (WHO) has reported several factors that may hinder pregnant women from accessing skilled antenatal and intrapartum care. These include; poverty, distance to a health facility, lack of information of where to access services, inadequate services and cultural practices [1].

Previous studies undertaken in rural Tanzania have also reported socio-cultural factors as one of the main determinants of maternal preference regarding place of birth [9]. For example, a socio-cultural tradition in some of the tribal cultures in Tanzania requires that when a married woman becomes pregnant for the first time, she has to go back to her parent’s home and the decisions of where to deliver rest on her mother [10].

Home delivery is a risk factor for the health of both a mother and a newborn [7]. In the effort of promoting hospital delivery, the government of Tanzania has established a program of building one health facility per village to address the challenge of walking distance to a nearby health facility [11]. The government of Tanzania has also removed the financial barriers to maternity services by removing out of pocket cost-sharing for delivering services [12]. It is stated in the health policy that all direct costs associated with pregnancy care and childbirth are to be covered by the government. The ultimate goal here is to remove financial barriers to accessing maternal health care services. On top of that, there are community campaigns by both government and non-governmental organizations to encourage utilization of health facility for delivery [13].

Despite all of these efforts, a large proportion of women in Tanzania and other developing countries continue to choose home birth over health facility. This study was conceptualized to explore the determinants of choice of home childbirth over health facility childbirth among women of reproductive age in Tanzania.

## Methods

### Study design and data

This study is a cross-sectional analysis of a dataset from the 2015-16 Tanzania Demographic and Health Survey and Malaria Indicator Survey (2015-16 TDHS-MIS).

### The 2015-16 TDHS-MIS

This section of the method has been published previously in the report of “Tanzania Demographic and Health Survey and Malaria Indicator Survey 2015-16” [2].

The 2015-16 TDHS-MIS is the ninth in a series of national sample surveys conducted in Tanzania to measure levels, patterns, and trends in demographic and health indicators. The survey was undertaken by the National Bureau of Statistics (NBS) and the Office of Chief Government Statistician (OCGS), Zanzibar, in collaboration with the Ministry of Health, Community Development, Gender, Elderly, and Children on the Tanzania Mainland and the Ministry of Health, Zanzibar. The primary objective of the 2015-16 TDHS-MIS was to provide up-to-date estimates of basic demographic and health indicators. The survey collected information on fertility levels, marriage, sexual activity, fertility preferences, awareness and use of family planning methods, breastfeeding practices, nutrition, childhood and maternal mortality, maternal and child health, malaria, and other health-related issues.

The sample design for the 2015-16 TDHS-MIS was done in two stages and was intended to provide.

estimates for the entire country, for urban and rural areas in Tanzania Mainland, and for Zanzibar. The first stage involved selecting sample points (clusters), consisting of enumeration areas (EAs) delineated for the 2012 Tanzania Population and Housing Census. A total of 608 clusters were selected. In the second stage, a systematic selection of households was involved. A complete households listing was carried out for all 608 selected clusters prior to the fieldwork. From the list, 22 households were then systematically selected from each cluster, yielding a representative probability sample of 13,360 households for the 2015-16 TDHS-MIS. All women age 15–49 who were either usual residents or visitors in the household on the night before the survey were included in the 2015-16 TDHS-MIS and were eligible to be interviewed. Out of a total of 13,360 households selected for the 2015-16 TDHS, 12,767 were occupied. Of the occupied households, 12,563 were successfully interviewed, yielding a response rate of 98%. In the interviewed households, 13,634 eligible women were identified for individual interviews; interviews were completed with 13,266 women, yielding a response rate of 97%.

Four questionnaires based on the DHS program’s standard were used for the 2015-16 TDHS-MIS: The Household Questionnaire, the Woman’s Questionnaire, the Man’s Questionnaire, and the Biomarker Questionnaire. In particular, the Woman’s Questionnaire was used to collect information from all eligible women age 15–49. The information collected includes background characteristics, birth history and childhood mortality, knowledge and use of family planning methods, fertility preferences, antenatal, delivery, and postnatal care, breastfeeding and infant feeding practices, vaccinations and childhood illnesses, marriage and sexual activity, women’s work and husbands’ background characteristics, adult mortality, including maternal mortality, malaria, domestic violence, and other health issues.

### Study population and data extraction

For the present analysis, a subset of the original TDHS-MIS dataset was abstracted using the following criteria: women of reproductive age (15–49 years) who gave birth within the year preceding the survey. The individual recodes (TZIR7BFL) file was used. The final sample size for this analysis resulted into 2,286 women. We then dropped unnecessary variables to this study from the data file.

### Study variables

Through a literature review, a conceptual framework was developed to guide the data review. The conceptual framework defined the primary independent variables (socio-demographic and obstetric characteristics of a woman), the intermediate variable (antenatal services utilization, coded 1 for adequate antenatal services utilization [four or more antenatal care visits] and coded 0 for inadequate antenatal services utilization [less than four antenatal care visits]), and the original outcome variable (place of delivery, later dichotomized into a dummy variable coded 1 if a woman delivered outside of a health facility, such as home or TBA premises, and 0 if she delivered at health facility).

### Data analysis

Data were analyzed using Statistical Package for Social Sciences (IBM SPSS version 20). Data analysis started by describing all study variables using frequencies and percentages. We then assessed the association between dependent variables and independent variables using the chi-squared test, and finally, we performed binary logistic regression analysis (univariate and multivariate) to determine significant predictors of the choice of place of delivery. All analyses were based on a 5% level of significance.

## Results

### Socio-demographic characteristics

The majority of women, *n* = 1549, (67.8%), were aged between 20 and 34 years, resided in rural Tanzania, *n* = 1767, (77.3%), had a primary level of education, *n* = 1357, (59.4%), had inadequate antenatal visits, *n* = 1262, (55.2%), and were married, *n* = 2003, (87.6%), (Table 1).

**Table 1:** Socio-demographic characteristics of study respondents

Variable	Frequency	Percentage
**Age groups(years)**		
15–19	324	14.2
20–34	1549	67.8
35–49	413	18.1
**Place of residence**		
Urban	519	22.7
Rural	1767	77.3
**Level of education**		
No formal education	454	19.9
Primary education	1357	59.4
Secondary+	475	20.8
**Wealth index**		
Poorest	555	24.3
Poorer	476	20.8
Middle	405	17.7
Richer	477	20.9
Richest	373	16.3
**Number of antenatal visits during pregnancy**		
0–3	1262	55.2
4+	1021	44.7
**Parity**		
Para One	589	25.8
Para 2–4	1015	44.4
Para 5+	682	29.8
**Owns a mobile telephone**		
No	1265	55.3
Yes	1021	44.7
**Marital Status**		
Never Married	186	8.1
Married	2003	87.6
Separated	97	4.2
**Respondent currently working**		
Not working	612	26.8
Working	1674	73.2
**Zones**		
Western	212	9.3
Northern	186	8.1
Central	233	10.2
Southern Highlands	145	6.3
Southern	69	3
South West Highlands	271	11.9
Lake	665	29.1
Eastern	180	7.9
Zanzibar	325	14.2

### Place of birth

The majority of study respondents, *n* = 1481, (64.8%), had health facility childbirth while a total of 805, (35.2%) women had home childbirth.

### Relationship between socio-demographic and obstetric characteristics with choice of place of childbirth

Socio-demographic characteristics that showed a significant relationship with the place of birth included: place of residence, *p* < 0.001; age group, *p* = 0.005; education level, *p* < 0.001; current marital status, *p* = 0.001; zones, *p* < 0.001; working status, *p* = 0.033, wealth index *p* < 0.001 and ownership of mobile phone, *p* < 0.001. The obstetric characteristics that showed a significant relationship were: number of antenatal visits, *p* < 0.001, and parity *p* < 0.001 (Table 2).

**Table 2:** The relationship between maternal services utilization during pregnancy and choice of place of childbirth

Variables	Health Facility	Home	X^**2**^	*p*-value
	n (%)	n (%)		
**Place of residence**				
Urban	448(86.3)	71(13.7)		
Rural	1033(58.5)	734(41.5)	136.479	<0.001
**Age group**				
15-19	227(70.1)	97(29.9)		
20-34	1011(65.3)	538(34.7)		
35-49	243(58.8)	170(41.2)	10.516	0.005
**Educational level**				
No education	201(44.3)	253(55.7)		
Primary education	882(65)	475(35)		
Secondary+	398(83.8)	77(16.2)	158.952	<0.001
**Parity**				
Para one	455(77.2)	134(22.8)		
Para 2-4	679(66.9)	336(33.1)		
Para 5+	347(50.9)	335(49.1)	99.897	<0.001
**Owns a mobile telephone**				
Not owning	696(55)	569(45)		
Owning	785(76.9)	236(23.1)	118.404	<0.001
**Wealth index**				
Poorest	256(46.1)	299(53.9)		
Poorer	250(52.5)	226(47.5)		
Middle	246(61)	158(39)		
Richer	380(79.7)	97(20.3)		
Richest	348(93.3)	25(6.7)	297.847	<0.001
**Number of antenatal visits during pregnancy**				
0-3	729(57.8)	533(42.2)		
4+	749(73.4)	271(26.6)	59.441	<0.001
**Marital Status**				
Never Married	146(78.5)	40(21.5)		
Married	1272(63.5)	731(36.5)		
Separated	63(64.9)	34(35.1)	16.764	<0.001
**Respondent currently working**				
Not working	418(68.3)	194(31.7)		
Working	1063(63.5)	611(36.5)	4.526	0.033
**Zones**				
Western	119(56.1)	93(43.9)		
Northern	134(72)	52(28)		
Central	147(63.1)	86(36.9)		
Southern Highlands	134(92.4)	11(7.6)		
Southern	60(87)	9(13)		
South West Highlands	168(62)	103(38)		
Lake	352(52.9)	313(47.1)		
Eastern	154(85.6)	26(14.4)		
Zanzibar	213(65.5)	112(34.5)	150.926	<0.001

### Determinants for choice of home birth over health facility birth

After adjusting for confounders, the determinants of home birth were: the level of education (primary education, [AOR = 0.666, *p* = 0.001]; secondary and higher education, [AOR = 0.417, *p* < 0.001]); not owning a mobile phone, (AOR = 1.312, *p* = 0.018); parity (para 2–4, [AOR = 1.594, *p* = 0.004], para 5 and above, [AOR = 2.158, *p* < 0.001]); inadequate antenatal visits, (AOR = 1.406, *p* = 0.001); wealth index (poorest, [AOR = 9.395, *p* < 0.001]; poorer, (AOR = 7.701, *p* < 0.001); middle, (AOR = 5.961, *p* < 0.001); richer, (AOR = 2.557, *p* < 0.001), and Zones (Southern Highlands, [AOR = 0.189, *p* < 0.001]; Southern, [AOR = 0.225, *p* < 0.001]; Zanzibar, [AOR = 2.55, *p* < 0.001]) (Table 3).

**Table 3:** Determinants of choice for home over health facility childbirth

Variable	OR	95% CI		***p***-value	AOR	95% CI		***p***-value
		Lower	Upper			Lower	Upper	
**Place of residence**								
Rural(ref)	1				1			
Urban	0.223	0.171	0.291	<0.001	0.774	0.55	1.09	0.142
**Age group**								
15-19(ref)	1				1			
20-34	1.245	0.96	1.615	0.098	1.134	0.795	1.617	0.488
35-49	1.637	1.203	2.228	0.002	0.886	0.555	1.414	0.611
**Level of educational**								
No education(ref)	1				1			
Primary education	0.428	0.345	0.531	<0.001	0.666	0.523	0.85	0.001
Secondary+	0.154	0.113	0.209	<0.001	0.417	0.284	0.614	<0.001
**Parity**								
Para one	1				1			
Para 2-4	1.68	1.331	2.121	<0.001	1.594	1.158	2.193	0.004
Para 5+	3.278	2.568	4.185	<0.001	2.158	1.47	3.168	<0.001
**Owns a mobile telephone**								
No	2.719	2.265	3.265	<0.001	1.312	1.048	1.643	0.018
Yes	1				1			
**Antenatal visits**								
4+	1				1			
0-3	2.013	1.685	2.406	<0.001	1.406	1.146	1.724	0.001
**Wealth index**								
Poorest	16.258	10.483	25.214	<0.001	9.395	5.435	16.24	<0.001
Poorer	12.584	8.073	19.615	<0.001	7.701	4.48	13.24	<0.001
Middle	8.904	5.665	13.997	<0.001	5.961	3.5	10.15	<0.001
Richer	3.553	2.236	5.646	<0.001	2.557	1.555	4.206	<0.001
Richest	1				1			
**Working status**								
Not working (ref)	0.807	0.663	0.983	0.034	0.979	0.767	1.248	0.861
Working	1				1			
**Marital Status**								
Never Married	1				1			
Married	2.098	1.461	3.011	<0.001	0.696	0.351	1.379	0.299
Separated	1.97	1.143	3.395	0.015	0.721	0.387	1.344	0.303
**Zone**								
Western	1				1			
Northern	0.497	0.326	0.755	0.001	1.062	0.657	1.718	0.805
Central	0.749	0.512	1.095	0.135	1.001	0.656	1.529	0.994
Southern Highlands	0.105	0.054	0.206	<0.001	0.189	0.093	0.384	<0.001
Southern	0.192	0.091	0.407	<0.001	0.225	0.103	0.493	<0.001
South West Highlands	0.784	0.544	1.131	0.193	0.842	0.564	1.259	0.403
Lake	1.138	0.834	1.553	0.416	1.4	0.993	1.973	0.055
Eastern	0.216	0.132	0.355	<00.1	0.573	0.328	1.002	0.051
Zanzibar	0.673	0.472	0.959	0.029	2.55	1.601	4.061	<0.001

## Discussion

This study was conducted to provide information on determinants of choice of home childbirth over health facility childbirth among women of reproductive age in Tanzania. Women who choose home birth may have an increased risk of maternal and neonatal morbidity and mortality. The government of Tanzania has invested on promoting the utilization of health facilities for childbirths as one of the strategies to decrease maternal and neonatal morbidity and mortality. Removal of financial barriers, increasing number of health facilities and increasing skilled human resources for health care are among the strategies that have been employed by the government to promote the utilization of health facilities for birth [14]. With all these efforts, one wonders why pregnant women still deliver at home where there is a very low possibility of being assisted by skilled birth attendants. This study used national survey data in order to provide an increased understanding of determinants of choice for home childbirth over health facility childbirth among women of reproductive age in the country.

This study revealed education level of a woman, parity, ownership of mobile phone, number of antenatal visits and geographic zone as the determinants of choice for place of childbirth among women of reproductive age in Tanzania.

The findings show that the odds of a pregnant woman having home birth over health facility birth decreases as a woman’s education level increased. Pregnant women with primary level education and secondary level education were 33% and 58%, less likely respectively to choose home for childbirth over health facility when compared to women with no formal education. A possible explanation could be, advancement in education level increased exposure to health information and knowledge as well as an increased income. Studies based on population surveys in Ethiopia had reported similar findings [15, 16].

This study also revealed that pregnant women who had higher parity were more likely to choose home childbirth over health facility childbirth. Pregnant women who had two to four children and those who had five or more children were 1.5 and 2.2 times more likely respectively to have a homebirth compared to primiparous women. Similar findings were reported in a study done in Ethiopia [15]. A history of uneventful birth which occurred outside a health facility could explain the mothers’ lowered risk perceptions of childbirth [7]. This suggests that women who had several births at home assisted by traditional birth attendants without complications would be more likely to utilize the same attendants for subsequent births. They may think that births assisted by unskilled birth attendants are not risky because of their personal history of uneventful births with these attendants. On the contrary, studies indicate that the risk of obstetric complications increases with each additional pregnancy [17]. Many symptoms of such complications may remain unnoticed until late in the process of delivery or even post-delivery.

Pregnant women who had access to mobile phones were more likely to use health facilities for childbirth than those without mobile phones. Essentially, women with access to mobile phones have a higher likelihood of receiving reproductive health information through their mobile devices when compared to those who have no access to phones.

The study indicated that there was a significant relationship between wealth index status and choice of place of delivery. women of low economic status were more likely to choose home for childbirth. Even though the government of Tanzania has removed out of pocket payment for childbirth services, there are other many costs associated with health facility childbirth. For example, transport costs for women in labor and the relatives who accompany them to a health facility may be a challenge in lower income families and may influence pregnant women to opt for home childbirth. There are also costs needed to prepare birth items requested by health care providers. If pregnant women in the course of pregnancy fail to gather the items needed for childbirth, they may decide to opt for home childbirth to avoid embarrassment in the health facility. The income factor has been reported by similar studies done elsewhere in low resources countries [18, 19].

The study also suggests that attendance at an adequate number of antenatal visits predicted the choice of place of delivery. Pregnant women who had inadequate antenatal visits were 1.4 times more likely to opt for home birth than those who had adequate antenatal visits. Although the majority of pregnant women in the survey had at least one antenatal visit, only 44.7% had the recommended four or more antenatal visits in Tanzania. Similar findings have been reported in similar studies done in Ethiopia [14] and Kenya [18]. The possible explanation could be that a pregnant woman who had adequate antenatal visits had more opportunities to learn about obstetric complications and their danger signs which could increase their perception of the risks associated with childbirth.

The study used geographical zones as a variable in determining choice of place of childbirth in order to ascertain the differences in use of home birth by residential location. Zones in Tanzania are used by national surveys to facilitate data collection. Our study found that zones predicted the place of childbirth. Women from Southern, Southern highlands and Eastern zones were less likely to choose home birth over health facility birth when compared with women from the Western zone. Women from Zanzibar had higher odds of choosing home birth over health facility birth when compared to pregnant women from the Western zone. Based on the significant differences in use of health facilities for childbirth among these zones, we recommend further studies to explore the possible factors among zones that could have contributed to women’s choice of location for childbirth.

Our study results suggest that additional innovative strategies may be needed to address the challenge of mothers choosing to have home births assisted by unskilled birth attendants. More efforts such as community sensitization to maternal services utilization and increased access to maternal services may need to be directed to the Western zone and especially to Zanzibar, which showed the most significant levels in maternal choice of home-birth.

Some studies from Tanzania have reported a lack of collective decision-making within households when choosing place of childbirth [9, 20]. Decision-making appears to be influenced by the gender division of roles and responsibilities: the pregnant woman, her mother and mother in-law have to decide the place of childbirth, while the men have the responsibility of providing money [9]. Men in Tanzania, like other low-income countries, have dominative power and control over the family economy [21]. Thus, including men in deciding the place of childbirth may increase utilization of health facility deliveries, if the free maternity services and the economic impact of maternal mortality on the household are taken into account, thence raising the number of births attended by skilled birth attendants.

This study was limited by its exclusive use of quantitative methods, which work to determine associations between variables, but do little to explain them. As such, a corresponding study using qualitative methodology for a more narrative overview of the findings might be useful to explore the research question in greater depth. Furthermore, the study, by nature of being a population survey, cannot prove a causal relationship between variables; it can only posit a connection. More research is needed to confirm and explore this topic.

## Conclusions

There are large proportions of women who choose home childbirth, most being assisted by unskilled birth attendants, mainly traditional birth attendants, relatives and friends. We found that the predictors for choosing home-based childbirth in Tanzania included illiteracy among women, poor access to mobile communication, inadequate antenatal visits, low socio-economic status and residence in Zanzibar. Based on the data and findings, the authors provide the following three recommendations: First, education and awareness programs should be designed to emphasize the importance of delivering at health facilities and the risks associated with delivering at home or outside of health facilities. Secondly, innovative interventions targeting women with the specific indicators listed above are needed to increase the use of health facilities for childbirth and hence reduce maternal and neonatal mortalities in Tanzania. Thirdly, maternal health services at all levels of health facilities, including dispensaries and health centers, should be improved and be friendly to users. This will motivate women, particularly those of low socioeconomic status, to utilize health facilities for delivery. Findings from this study may be useful in informing policy makers and public health experts in the area so as to improve maternal health outcomes by improving the utilization of health facilities by women during labor and delivery.

## Data Availability

The data that support this analysis are available at https://dhsprogram.com/data/ subject to permission from MEASURE DHS.

## Abbreviations

AOR: Adjusted Odds Ratio
DHS: Demographic Health Survey
MoHCDEC: Ministry of Health, Community Development, Gender, Elderly and Children
NBS: National Bureau of Statistics
TBA: Traditional Birth Attendant
TDHS-MIS: Tanzania Demographic and Health Survey and Malaria Indicator Survey
USAID: United States Agency for International Development
WHO: World Health Organization

## References

[1] WHO. Monitoring Health for the SDGs [Internet]. Vol. 8, Αγαη. 2019. Available from: https://apps.who.int/iris/bitstream/handle/10665/324835/9789241565707-eng.pdf.

[2] MoHCDGEC. Tanzania Demographic and Health Survey and Malaria Indicator Survey [Internet]. Ministry of Health, Community Development, Gender, Elderly and Children (MoHCDGEC). Dar es Salaam, Tanzania, and Rockville, Maryland, USA; 2015. Available from: https://www.dhsprogram.com/pubs/pdf/FR321/FR321.pdf.

[3] UN. The sustainable development goals report 2016. Sustain Dev goals Rep 2016 [Internet]. 2016; Available from: https://unstats.un.org/sdgs/report/2019/The-Sustainable-Development-Goals-Report-2019.pdf.

[4] Bwana VM, Rumisha SF, Mremi IR, Lyimo EP, Mboera LEG. Patterns and causes of hospital maternal mortality in Tanzania A 10-year retrospective analysis. Available from: https://journals.plos.org/plosone/article?id=10.1371/journal.pone.0214807. 10.1371/journal.pone.0214807 PMC6456219 30964909

[5] Hug L, Sharrow D, Sun Y, Marcusanu A, You D, Mathers C, et al. levels and Trends in Child Mortality. 2017; Available from: http://www.childmortality.org/files_v21/download/IGME report 2017 child mortality final.pdf.

[6] World Health Organization, United Nations Population Fund. United Nations Children’s Fund, International Confederation of Midwives, International Confederation of Nurses, International Federation of Gynecology and Obstetrics, et al. Definition of skilled health personnel providing care during childbirth. 2018;1–4. Available from: https://apps.who.int/iris/bitstream/handle/10665/272818/WHO-RHR-18.14-eng.pdf?ua=1.

[7] Mahiti GR, Kiwara AD, Mbekenga CK, Hurtig A-K, Goicolea I. “We have been working overnight without sleeping”: traditional birth attendants’ practices and perceptions of post-partum care services in rural Tanzania. BMC Pregnancy Childbirth [Internet]. 2015;15(1):8. Available from: http://www.scopus.com/inward/record.url?eid=2-s2.0-84924213233&partnerID=tZOtx3y1. 10.1186/s12884-015-0445-z PMC4324777 25643622

[8] Vyagusa DB, Mubyazi GM, Masatu M (2013). Involving traditional birth attendants in emergency obstetric care in Tanzania: Policy implications of a study of their knowledge and practices in Kigoma Rural District. Int J Equity Health.

[9] Moshi F, Nyamhanga T. Understanding the preference for homebirth; an exploration of key barriers to facility delivery in rural Tanzania. Reprod Health [Internet]. 2017;14(1):132. Available from: http://reproductive-health-journal.biomedcentral.com/articles/10.1186/s12978-017-0397-z. 10.1186/s12978-017-0397-z PMC5645928 29041972

[10] Lyimo FS, Beran TN. Demographic, knowledge, attitudinal, and accessibility factors associated with uptake of cervical cancer screening among women in a rural district of Tanzania: Three public policy implications. BMC Public Health [Internet]. 2012;12(1):22. Available from: http://bmcpublichealth.biomedcentral.com/articles/10.1186/1471-2458-12-22. 10.1186/1471-2458-12-22 PMC3299640 22233530

[11] Ministry of health and social welfare and prime Minister’s ofice regional ad ministritation and local government. Summary and Analysis of the Comprehensive Council Health Plans 2013/2014 [Internet]. 2013. Available from: http://www.tzdpg.or.tz/fileadmin/documents/dpg_internal/dpg_working_groups_clusters/cluster_2/health/JAHSR-2013/Summary_and_Analysis_of_CCHP_2013-2014_Report.pdf.

[12] The United Republic of Tanzania Ministry of Health and Social Welfare. National Health Policy. Minist Heal. 2003;1–37.

[13] Manzi F, Daviaud E, Schellenberg J, Lawn JE, John T, Msemo G (2017). Improving Newborn Survival in Southern Tanzania (INSIST) trial; Community-based maternal and newborn care economic analysis. Health Policy Plan.

[14] MoHSW. Tanzania Health Sector Strategic Plan 2015–2020 (HSSP IV). Vol. 2020. 2015.

[15] Fekadu M, Regassa N (2014). Skilled delivery care service utilization in Ethiopia: Analysis of rural-urban differentials based on national demographic and health survey (DHS) data. Afr Health Sci.

[16] Tukur D, Oche O. Determinants of antenatal care, institutional delivery and postnatal care services utilization in Nigeria. 2015;1–17. 10.11604/pamj.2015.21.321.6527 PMC4633744 26587168

[17] Al-Shaikh GK, Ibrahim GH, Fayed AA, Al-Mandeel H (2017). Grand multiparity and the possible risk of adverse maternal and neonatal outcomes: A dilemma to be deciphered. BMC Pregnancy Childbirth.

[18] Nyongesa C, Xu X, Hall JJ, Macharia WM, Yego F, Hall B (2018). Factors influencing choice of skilled birth attendance at ANC: Evidence from the Kenya demographic health survey. BMC Pregnancy Childbirth.

[19] Amoakoh-Coleman M, Ansah EK, Agyepong IA, Grobbee DE, Kayode GA, Klipstein-Grobusch K (2015). Predictors of skilled attendance at delivery among antenatal clinic attendants in Ghana: A cross-sectional study of population data. BMJ Open.

[20] Kohi TW, Mselle LT, Dol J, Aston M (2018). When, where and who? Accessing health facility delivery care from the perspective of women and men in Tanzania: A qualitative study. BMC Health Serv Res.

[21] United Nation. Men in families [Internet]. Vol. 7, Marriage and Family Review. 1984. 143–162 p. Available from: https://www.un.org/esa/socdev/family/docs/men-in-families.pdf.

